# Assessing Phonological Profiles in Children and Adolescents With Down Syndrome: The Effect of Elicitation Methods

**DOI:** 10.3389/fpsyg.2021.662257

**Published:** 2021-05-12

**Authors:** Eliseo Diez-Itza, Patricio Vergara, María Barros, Manuela Miranda, Verónica Martínez

**Affiliations:** ^1^LOGIN Research Group, Department of Psychology, University of Oviedo, Oviedo, Spain; ^2^School of Speech-Language Pathology, Austral University of Chile, Puerto Montt, Chile

**Keywords:** Down syndrome, phonological profiles, elicitation methods, atypical language development, neurodevelopmental disorders

## Abstract

In the context of comparing linguistic profiles across neurodevelopmental disorders, Down syndrome (DS) has captured growing attention for its uneven profile. Although specific weaknesses in grammatical and phonological processing have been reported, research evidence on phonological development remains scarce, particularly beyond early childhood. The purpose of this study was to explore the phonological profiles of children and adolescents with Down syndrome. The profiles were based on the frequency and relative proportion of the processes observed by classes, and they were compared to those of typically developing preschool children of similar verbal age. A complementary goal was to assess the effect of two different methods of elicitation: a test of articulation and spontaneous speech sampling. Finally, intergroup and intragroup differences in full match percentages between three positions at syllable-level (complex onset, medial coda, and final coda) were assessed. The results of the present study confirmed that the frequency of phonological processes in children and adolescents with DS is atypically high and is above what is expected for lexical age and at the same level as grammatical age. Highly increased frequency of processes, consistent in all kinds of processes and positions at the syllable-level, and asynchronous with verbal age and mental age suggest atypical developmental trajectories of phonological development in the Down syndrome population.

## Introduction

Down syndrome (DS) is a neurodevelopmental genetic disorder, caused by trisomy of the chromosome 21, and the most common cause of intellectual disability. Individuals with DS share a unique neuropsychological profile, characterized by a complex pattern of strengths and weaknesses, which was partially unveiled when compared with other neurodevelopmental disorders like Williams syndrome (WS) or fragile X syndrome (FXS) (Bellugi et al., 2000; Roberts et al., 2008; Grieco et al., 2015). However, developmental emergence of the neurocognitive profile and later trajectories of development in childhood and adolescence are not yet well-explored and understood (Channell et al., 2014). Linguistic skills are particularly impaired in individuals with DS so they consistently tend to show better performance on non-verbal tasks than on verbal tasks (Næss et al., 2011). Different asymmetries are also observed in the linguistic profile: comprehension is better than expression, and relative strengths in early vocabulary and pragmatics of social communication contrast with relative weaknesses in morphosyntactic production and phonological processing (Chapman, 2006; Diez-Itza et al., 2019). Although grammar is often pointed out as the most impaired linguistic domain in DS, studies underscore the importance of its relationship with phonological disorders, which in turn have serious implications for communication by generating significant levels of unintelligibility (Kumin, 2006; Christodoulou, 2015). Across languages, including for Spanish, studies report both universal and language-specific patterns in typical and protracted phonological development (Bernhardt and Stemberger, 2017).

As in other domains, the debate about delayed or atypical language development has been raised in DS phonological research (Thomas and Karmiloff-Smith, 2003; Levy and Eilam, 2013). After a late onset of language in toddlers with DS, it is discussed whether later development follows the same pathway as typical development at a slower rate (normalcy approach) or presents atypical characteristics (neuroconstructivist approach) (Thomas and Karmiloff-Smith, 2003; Schaner-Wolles, 2004; Levy and Eilam, 2013).

Interpretation of research results adopt the delay approach in several studies. Van Borsel (1988, 1996) observed that children, adolescents and adults with DS and TD pairs showed similar phonological processes (cluster reduction, deletion of final consonants, deletion of unstressed syllables, and consonant substitutions like fronting or devoicing) and types of errors: omissions, substitutions (place, manner, or voice), and distortions (nasalized, denasalized, lateralized, aspirated, labialized, fronted, backed, devoiced, voiced). In the same vein, Parsons and Iacono (1992) found no evidence to support the claim of a unique phonological profile in children and adolescents with DS. They showed a greater number of errors that are characteristic of both TD children and phonologically-impaired populations but not specific of DS. Children with DS in the study of Yousif (2018), despite a delayed onset and a slower pace of phonological development, appeared to develop phonology in a similar way to TD children. When compared with FXS, ASD, and TD children, participants with DS presented the same classes of phonological processes (consonant omission or substitution, syllable deletion, and assimilation) than their TD MA-matched pairs (Barnes et al., 2009).

The disordered approach claims that inconsistent error patterns, asynchronous phonological development, and production of rare processes in late development, suggest atypical profiles in individuals with DS emerging early on the transition to first words (Dodd, 1976; Roberts et al., 2007; Martin et al., 2009). Nevertheless, inconsistent production of words appears to be different from children with phonological disorder (Dodd and Thompson, 2001). In a study including FXS and TD children, Roberts et al. (2005) found greater speech delays in the children with DS and a differentiated phonological profile with low percentages of word full match. Hidalgo de la Guía and Garayzábal (2019) defined specific phonetic-phonological profiles for participants with DS, WS, and Smith-Magenis syndrome (SMS) based on segmental and syllable structure phonological analyses. Participants in the DS group showed a higher frequency of processes at both levels: segmental (simplification and omission of trills and fronting) and syllable structure (reduction on complex onsets and nuclei and omission of codas). Atypical processes more frequent in the DS group were tap and trills backing, deaffrication, and labial assimilation.

Atypical characteristics of the phonological profile in DS could be related to working memory (Baddeley and Jarrold, 2007), and specifically to a phonological loop specific deficit associated with the absence of spontaneous rehearsal and reduced storage capacity (Jarrold et al., 2000; Chapman and Hesketh, 2001; Laws and Gunn, 2004; Vicari et al., 2004).

Assessments of phonological development in DS populations are mainly based on two elicitation methods: tests of articulation (Dodd, 1976; Van Borsel, 1988, 1996; Roberts et al., 2005; Rupela et al., 2010); spontaneous or connected speech (Stoel-Gammon, 1980; Barnes et al., 2009); and sometimes on a combination of both an articulation test and spontaneous speech (Sommers et al., 1988; Parsons and Iacono, 1992; Yousif, 2018; Hidalgo de la Guía and Garayzábal, 2019). Studies indicate a higher frequency of phonological processes in connected speech, but only Yousif (2018) reports some evidence. However, the effects of speech sample sizes and repeated processes in the same word tokens need to be controlled. It has been suggested that articulation tests provide sufficient and representative information for phonological assessment although phonotactic probability and word length should be controlled (Masterson et al., 2005; Edwards and Beckman, 2008). However, the validity of conversational speech samples providing a wider analytical perspective for optimal measures of speech performance has also been underscored (Shriberg and Kwiatkowski, 1980, 1985; Morrison and Shriberg, 1992).

### Objectives

The main objective of the present study is to explore the phonological profiles of a group of Spanish-speaking children and adolescents with Down syndrome, and to compare them with those of a group of typically developing preschool children of similar verbal age range. In order to fulfill the additional objective of comparing methods of phonological assessment, the oral production samples of words for the analyses were obtained using two elicitation methods: a test of articulation and spontaneous speech. Based on previous studies, it is hypothesized that frequency of phonological processes will be lower than expected for verbal age in the Down syndrome group and that the articulation test will be more demanding (i.e., sensitive) due to increased complexity of targets.

The specific objectives are: (1) to compute frequency of phonological processes and to compare intergroup differences in total frequency, and the profiles of frequency of processes by classes: Syllable structure, Substitution, Omission, Assimilation, and Addition; (2) to determine the effect of the assessment method on frequency of processes, and the effect of interaction between elicitation method and group; (3) to assess the profiles of relative frequency of processes (proportion) by class as a function of group and method, in search for atypical features in the phonological profile of individuals with Down syndrome; and (4) to analyze the differences in the proportion of matches at three positions at the syllable-level: complex onset (tautosyllabic consonant clusters), word-medial coda (C1 in heterosyllabic consonant clusters), and word-final coda.

## Method

### Participants

The participants were 24 children and adolescents with Down syndrome (Age-M: 13.9; range: 7 years; 1 month−19 years; 1 month; Verbal Age-M: 4.3; range: 3 years; 9 months−5 years; 9 months), clinically diagnosed and tested for full trisomy 21; and 52 typically developing preschool children (Age-M: 4.1; range: 3 years−5 years; 4 months); all of them were monolingual speakers of Spanish from middle-class backgrounds and gave informed consent to participate in the study. The criterion for inclusion in the DS group was a sufficient verbal ability to engage in conversation, and in the TD group it was the absence of language problems or protracted development. Verbal age in the DS group was calculated as a composite of lexical age, obtained from Peabody Vocabulary Test (PPVT: Lexical age-M: 5.8; range: 5 years−7 years; 11 months), and a grammatical age estimate based on MLU age stages (Levy and Eilam, 2013) (Grammatical age-M: 2.9; range: 2 years; 6 months−3 years; 6 months).

The Down syndrome group (DS) and the Typical Development group (TD) were divided into four subgroups by method of elicitation (AT: articulation test vs. SS: spontaneous speech): DS-AT (seven females/five males) (Age-M: 14.4; range: 10 years; 4 months−18 years; 9 months); DS-SS (seven females/five males) (Age-M: 13.5; range: 7 years; 1 month−19 years; 1 month); TD-AT (eight females/four males) (Age-M: 4.1; range: 3 years−5 years; 4 months); TD-SS (20 females/20 males) (Age-M: 4.2; range: 4 years−4 years; 4 months).

The TD-AT group was further divided into three age groups of four children: TD-AT3 (Age-M: 3.1; range: 3 years−3 years; 2 months); TD-AT4 (Age M: 4.1; range: 4 years−4 years; 3 months); and TD-AT5 (Age M: 5.2; range: 5 years−5 years; 4 months).

### Instruments and Procedures

Phonological assessment was based on the oral production of word types collected using two methods of elicitation: (1) a test of articulation (TA) (*Prueba de Fonolog*í*a en Español-PFE:* Bernhardt et al., 2016); (2) transcription and analysis of spontaneous speech (SS). The PFE was administered individually by two investigators in the context of speech therapy sessions and was audiotaped. During the test, if the children did not produce spontaneously a target-word after being shown the picture, they were given a choice between two words and, if they still failed, a direct repetition of the word was elicited in order to assess all the items. The PFE contains 100 items (pictures to name), including nouns and verbs of diverse length, stress, and syllable structure. They were transcribed in the *International Phonetic Alphabet* (IPA) (International Phonetic Association, 2018) and analyzed with the tools of the Phon Project (Rose and MacWhinney, 2014). Using the program Phon 3.1 (Hedlund and Rose, 2019) the words were introduced in independent records, building a corpus for each participant with the audio and the orthographic and phonetic transcripts of the words (IPA target and IPA actual). The investigators who administered the test made independent transcriptions with a high level of agreement (99.5%); discrepancies were solved by the principal investigator, a total of 995 processes were analyzed and only three words were considered unintelligible. The spontaneous speech samples were obtained from recorded dyadic conversations in naturalistic settings between the children and an investigator, and then transcribed and analyzed with the CLAN programs of the CHILDES Project (MacWhinney, 2000). A transcription agreement of 99.5% was reached following the same procedure as in the articulation test, a total of 3,736 processes were analyzed and 44 words were considered unintelligible.

### Data Analysis

The dependent variables of the study were: (i) Frequency of phonological processes: total and by classes; (ii) Relative distribution (percentage) of phonological processes by classes; (iii) Full match of phonemes (percentage) at different positions in syllable structure: complex onset, medial coda and final coda.

Phonological processes were analyzed and categorized into one of the following classes (Diez-Itza et al., 2001): Syllable structure, Substitution, Omission, Assimilation, and Addition. Syllable structure processes include patterns of substitution and omission in complex onsets, complex nuclei, and codas at the syllable-level; whole syllable omission; metathesis; coalescence; and epenthesis. Substitution and Omission processes refer only to patterns affecting consonants and vowels serving as simple onsets and simple nuclei.

In order to control for the covariant effect of the size of spontaneous speech samples, frequency of phonological processes (total and by classes) was expressed through a Phonological Processes Index (PPI) (number of processes over 100 word types), obtaining quantitative profiles of groups. In spontaneous speech samples, only processes affecting different word types were taken into account. Relative distribution of processes by classes in percentage was also calculated for each subgroup in order to obtain qualitative profiles of groups (i.e., independent of absolute frequency of processes), and to assess the possible atypical distribution of processes in the Down syndrome group. The program Phon 3.0 (Hedlund and Rose, 2019) provided percentages of Full match of phonemes at different positions in syllable structure: complex onsets, codas (medial and final).

Differences in frequency (PPI) and relative distribution (percentage) of processes (total and by classes) were assessed with 2 × 2 two-factor ANOVA's of group [DS,TD] × Method [AT,SS], including statistical power analyses. Independent one-factor ANOVA's were conducted to assess differences in frequency of processes between the DS-AT group and TD-AT age subgroups. One-factor multivariate ANOVA's of subgroups [DS-AT, DS-SS, TD-AT, TD-SS] allowed for *post-hoc* comparisons with Tukey's HSD. Intergroup and intragroup Full Match differences in position at syllable-level were assessed with independent samples *t*-tests. Effect size was estimated using Cohen's *d* calculated with the tools provided in Lenhard and Lenhard (2016).

## Results

Table 1 reports the results of the ANOVA's of two factors: group [Down syndrome, Typical Development] × method [Articulation test, Spontaneous speech], conducted to assess differences in frequency of processes as expressed by a Phonological Processes Index (PPI) (Total and by Classes of processes: Syllable structure, Substitution, Omission, Assimilation, and Addition). It also includes frequency of processes (PPI) means and standard deviations for each group (Down syndrome, Typical Development) and method (Articulation test, Spontaneous speech). ANOVA's report statistically significant results on total frequency of processes (PPI total) and in all classes of phonological processes.

**Table 1:** Phonological processes index (total and by classes) means and standard deviations for groups and methods, 2×2 ANOVA *F* values, effect size, and power of tests.

	**DS (*n* = 12/12)**	**TD (*n* = 12/40)**	***F***	***p***	***d***	**1–β**
*PPI* total	50.18 (24.40)	18.34 (20.88)				
Articulation test	54.50 (16.98)	28.41 (24.40)	13.27	0.000	1.48	1.00
Spontaneous speech	45.87 (30.26)	15.32 (19.01)				
*PPI* syllable structure	27.44 (13.76)	9.79 (11.52)				
Articulation test	27.08 (13.00)	15.16 (12.90)	12.52	0.000	1.45	1.00
Spontaneous speech	27.80 (15.05)	8.18 (10.73)				
*PPI* substitution	15.33 (9.54)	6.06 (8.66)				
Articulation test	22.08 (4.68)	8.58 (8.97)	13.24	0.000	1.49	1.00
Spontaneous speech	8.42 (8.15)	5.35 (8.55)				
*PPI* omission	3.86 (5.86)	0.76 (0.99)				
Articulation test	1.50 (1.78)	1.16 (1.03)	10.09	0.000	1.30	0.99
Spontaneous speech	6.23 (7.51)	0.64 (0.96)				
*PPI* assimilation	2.25 (1.96)	0.75 (0.97)				
Articulation test	2.50 (2.02)	1.25 (1.42)	7.75	0.000	1.14	0.98
Spontaneous speech	2.00 (1.96)	0.60 (0.75)				
*PPI* addition	1.36 (1.25)	0.93 (1.66)				
Articulation test	1.33 (1.56)	2.25 (2.93)	4.90	0.004	0.90	0.89
Spontaneous speech	1.40 (0.91)	0.53 (0.70)				

*PPI, Phonological processes index; DS, Down syndrome group; TD, Typical development group; d, Cohen's effect size; 1–β, Power of test*.

The ANOVA for Total frequency of processes showed a main effect of group [*F*_(1, 75)_ = 24.78; *p* < 0.001; *d* = 1.17], such that the Down syndrome group presented a significantly higher index of phonological processes. ANOVA's for the different classes of processes yielded the following main effects and interaction effects: for Syllable structure processes, a main effect of group [*F*_(1, 75)_ = 24.40; *p* < 0.001; *d* = 1.16], indicating that the frequency of Syllable structure processes was significantly higher in the DS group; for Substitution processes, a main effect of group [*F*_(1, 75)_ = 15.30; *p* < 0.001; *d* = 0.92], a main effect of method [*F*_(1, 75)_ = 15.90; *p* < 0.001; *d* = 0.94], and an interaction effect of group x method [*F*_(1, 75)_ = 6.07; *p* = 0.016; *d* = 0.58], which indicate that the frequency of Substitution processes was significantly higher in the DS group and in the Articulation test condition, and that the effect of method was greater in the DS group; for Omission processes, a main effect of group [*F*_(1, 75)_ = 13.09; *p* = 0.001; *d* = 0.85], a main effect of method [*F*_(1, 75)_ = 6.60; *p* = 0.012; *d* = 0.61], and an interaction effect of group x method [*F*_(1, 75)_ = 10.31; *p* = 0.002; *d* = 0.76], indicating that the frequency of Omission processes was significantly higher in the DS group and in the Spontaneous speech condition, and that the effect of method was greater in the Down syndrome group; for Assimilation processes, a main effect of group [*F*_(1, 75)_ = 14.06; *p* < 0.001; *d* = 0.88], which reveal that the frequency of Assimilation processes was significantly higher in the DS group; for Addition processes, a main effect of method [*F*_(1, 75)_ = 4.79; *p* = 0.032; *d* = 0.51], and an interaction effect of group x method [*F*_(1, 75)_ = 5.598; *p* = 0.021; *d* = 0.557], showing that the frequency of Addition processes was significantly higher in the Articulation test condition and that the effect of method was higher in the Typical development group.

Independent one-factor ANOVA's were conducted with the aim of analyzing differences in frequency of processes [PPI total] in the Articulation test condition between the Down syndrome group (M = 54.50; SD = 16.98) and the Typical Development age subgroups: 3 years (M = 57.50; SD = 15.80); 4 years (M = 21.25; SD = 8.38); and 5 years (M = 6.50; SD = 5.20). Significant differences were observed between the Down syndrome group and the subgroups of 4 years [*F*_(1, 15)_ = 13.72; *p* = 0.002; *d* = 1.98] and 5 years [*F*_(1, 15)_ = 29.74; *p* < 0.001; *d* = 2.92], indicating that frequency of processes in the Down syndrome group is similar to that found in three-year-old children. Thus, frequency of processes in the Down syndrome-Articulation test group is at the expected level for “grammatical age” (M = 2.9), but under the expected level for “lexical age” (M = 5.8).

Table 2 reports one-way Multivariate ANOVA of differences between groups in frequency of processes (total and by classes) and statistically significant Tukey's HSD *post-hoc* contrasts.

**Table 2:** Subgroup differences in frequency of processes (PPI) and HSD Tukey *post-hoc* contrasts.

	**DS vs. TD**			**AT vs. SS**		
**PPI**	**SS vs. SS**	**AT vs. AT**	**SS vs. AT**	**AT vs. SS**	**DS vs. DS**	**TD vs. TD**
Total	30.54^***^	26.08^*^	17.45	39.17^***^	8.62	−13.09
Syllable Structure	19.61^***^	11.91	12.63	18.89^***^	−0.72	6.97
Substitution	3.06	13.50^***^	−0.16	16.72^***^	13.66^***^	3.22
Omission	5.59^***^	0.33	5.07^***^	0.85	−4.73^**^	0.52
Assimilation	1.40^*^	1.25	0.75	1.89^***^	0.49	0.64
Addition	0.86	−0.91	−0.84	0.79	−0.06	1.71*^*^

*PPI, Phonological processes index; DS, Down syndrome group; TD, Typical development group; AT, Articulation test; SS, Spontaneous speech. CI: 95%;*

* *p ≤ 0.05;*

** *p ≤ 0.01;*

*** *p ≤ 0.001*.

To assess differences in relative frequency of processes (total and by classes), i.e., proportional distribution of processes in percentage terms, a multivariate ANOVA of two factors was conducted: group [Down syndrome, Typical Development] × method [Articulation test, Spontaneous speech]. One participant in the TD-AT group was not included because he did not present any process. ANOVA's for the different classes of processes yielded the following main effects and interaction effects: no main effects of group; for Substitution processes, a main effect of method [*F*_(1, 74)_ = 9.59, *p* = 0.003; *d* = 0.74], and an interaction effect of group x method [*F*_(1, 74)_ = 13.94, *p* < 0.001; *d* = 0.89], indicating higher percentage of Substitution processes in the Articulation test than in Spontaneous speech, and that the effect of method was higher in the DS group: for Syllable structure processes, an interaction effect of group x method [*F*_(1, 74)_ = 4.95, *p* = 0.029; *d* = 0.56], reflecting an increased proportion of Syllable structure processes in the Typical development group.

**Figure 1:**
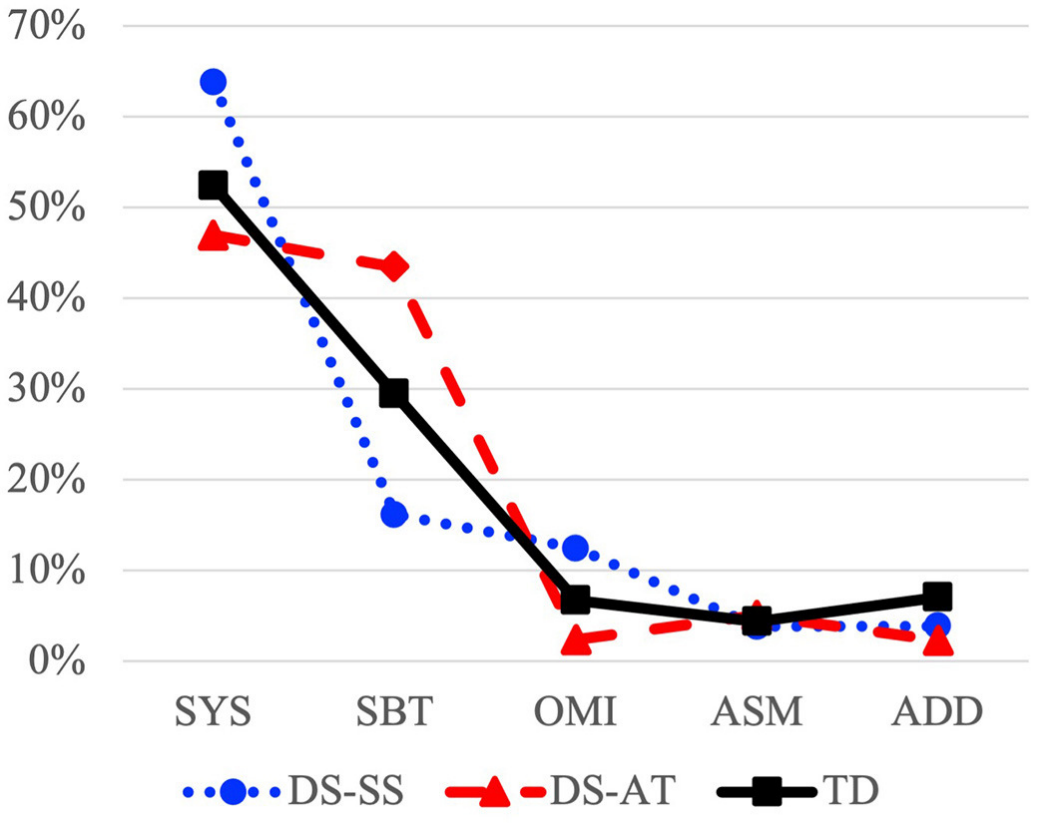
Profiles of relative frequency of processes (in percentages) by classes (SYS, Syllable Structure; SBT, Substitution; OMI, Omission; ASM, Assimilation; ADD, Addition) for DS subgroups (DS-SS, Spontaneous Speech; DS-AT, Articulation Test) and TD group.

Figure 1 represents the profiles of relative frequency of processes by classes (in percentages) for each group. It shows both DS subgroups independently (DS-AT and DS-SS) while the whole TD group is represented, since *post-hoc* subgroup comparisons with Tukey's HSD did not show differences between TD subgroups (TD-AT and TD-SS). A one-factor ANOVA with the three groups (TD, DS-AT, and DS-SS) was then carried out and revealed differences between the groups only in the proportion of Substitution processes [*F*_(2, 74)_ = 10.00; *p* < 0.001; *d* = 1.05]. *Post-hoc* subgroup comparisons with Tukey's HSD showed significant differences in the proportion of Substitution processes between the TD group and both DS-AT subgroup (difference of Means = −13.2; *p* = 0.020) and DS-SS subgroups (difference of Means = 14.0; *p* = 0.012); significant differences in proportion of Substitution processes were also found between DS subgroups (difference of Means = 27.3; *p* < 0.001). Furthermore, a tendency was observable, that the DS-SS subgroup presented a higher proportion of Syllable Structure and Omission processes than the TD group in line with absolute frequency (PPI) results; that the DS-AT subgroup presented a lower proportion of Syllable Structure and Omission processes than the TD group; that proportion of Assimilation processes was similar in the three groups; and that proportion of Addition processes was lower in both DS subgroups than in the TD group.

Table 3 reports means and standard deviations of Full Match (in percentage) in complex onsets and coda positions (Medial codas, Final codas), and the results of independent samples *t*-tests conducted to assess differences between the DS group and the TD group in the Articulation test. There were significant differences in Full Match percentages both in Total and in each coda position, while the difference in complex onset positions failed to reach significance. Additional independent samples *t*-tests were conducted to assess intragroup differences in Full match percentage between positions (complex onsets, codas, medial codas, final codas). Within the Down syndrome group, the following significant differences between positions were observed: Complex onsets vs. Codas [*t*_(23)_ = −2.80; *p* = 0.017; *d* = 0.89]; Complex onsets vs. Medial codas [*t*_(23)_ = −1.96; *p* = 0.076; *d* = 0.47]; Complex onsets vs. Final codas [*t*_(23)_ = −3.13; *p* = 0.010; *d* = 1.28]; and Medial codas vs. Final codas [*t*_(23)_ = −3.21; *p* = 0.008; *d* = 1.03]. Similar differences were found within the TD group: Complex onsets vs. Codas [*t*_(23)_ = −3.03; *p* = 0.011; *d* = 0.79]; Complex onsets vs. Medial codas MCOD [*t*_(23)_ = −2.34; *p* = 0.039; *d* = 0.59]; Complex onsets vs. Final codas [*t*_(23)_ = 3.67; *p* = 0.004; *d* = 1.02]; and Medial codas vs. Final codas [*t*_(23)_ = −3.90; *p* = 0.002; *d* = 1.10]. These results suggest that syllable structure positions have an effect on phonological production in both groups: Full match is significantly lower in complex onset positions (i.e., it is the most difficult position), and it is significantly higher in Final coda positions than in Medial coda positions (i.e., final coda is the easiest position).

**Table 3:** Full match percentage in complex onset and coda positions, *t*-test values and Cohen's *d* effect sizes.

**Full match**	**DS (*n* = 12)**	**TD (*n* = 12)**	***t***	***p***	***d***
Complex onsets	51.5 (24.07)	66.7 (29.86)	1.37	0.184	0.56
Codas	70.2 (14.13)	87.6 (8.97)	3.61	0.002	1.47
Medial codas	61.6 (16.49)	82.0 (13.59)	3.31	0.003	1.35
Final codas	79.2 (17.58)	95.3 (4.10)	3.10	0.009	1.26
Total	63.8 (14.48)	80.6 (15.00)	2.80	0.010	1.14

*DS, Down syndrome group; TD, Typical development group; d, Cohen's effect size*.

## Discussion

The results of the present study confirmed that the frequency of phonological processes in children and adolescents with DS is atypically high and is above what is expected for lexical age and at the same level as grammatical age. However, the percentage distribution by classes of processes (syllable structure, substitution, omission, assimilation, and addition) was not significantly different from that observed in children with DT of similar verbal age. As for typical development, the most difficult position at syllable-level is complex onset while coda in final position is the easiest one. The elicitation method based on the articulation test generated a higher frequency of processes, but it did not seem to affect the profiles of children with typical development. Instead, it determined fewer syllable structure and more substitution processes in the children and adolescents with Down syndrome. Although the results are not conclusive about simply delayed vs. disordered phonological profiles, highly increased frequency of processes, asynchronous with verbal age and mental age, suggests atypical developmental trajectories of phonological development in the Down syndrome population. Future studies should combine methods and perform more detailed analyses of atypical phonological processes in DS, which is crucial to design interventions that improve social communication by increasing intelligibility.

The purpose of the study was to explore the phonological profiles of children and adolescents with Down syndrome. The profiles were based on the frequency and relative proportion of the processes observed by classes, and they were compared to those of typically developing preschool children of similar verbal age. A complementary goal was to assess the effect of two different methods of elicitation: a test of articulation and spontaneous speech sampling. Finally, intergroup and intragroup differences in full match percentages between three positions at syllable-level (complex onset, medial coda, and final coda) were assessed.

Profiles based on the assessment of frequency of processes over 100 word types (PPI) showed a significantly higher number of phonological processes in the DS group, and consequently phonological development would be below expectations for verbal age. This finding is consistent with previous research suggesting that phonology is an area of special weakness in the speech production of individuals with DS from early on in development (Kent and Vorperian, 2013). When assessed with the articulation test, frequency of processes was similar in the DS group and the subgroup of TD 3-year-olds, while it was significantly higher in the DS group than in both groups of older TD preschoolers (4- and 5-year-olds). Thus, phonological development in the DS group would be more in agreement with grammatical verbal age, which usually corresponds to a MLU <3 in that population (Diez-Itza et al., 2019), than with lexical verbal age. The present study suggests a phonological-lexical asynchronous development which could be partially explained by phonological memory deficits (Laws and Gunn, 2004). Beyond a global delay of language development in the early stages, Iverson et al. (2003) reported an additional delay in making the transition from one- to two-word speech as observed in grammatical development (Vicari et al., 2000). It appears that phonology fails to emerge at the expected rate in parallel with vocabulary growth (Jackson-Maldonado et al., 2019).

The profiles of frequency of processes by classes showed quantitative differences in all classes, except in Addition. These results were consistent with research reviewed by Stoel-Gammon (2001). A high frequency of substitution and syllable structure processes appears to be a characteristic of DS compared to other neurodevelopmental genetic disorders (Barnes et al., 2009; Hidalgo de la Guía and Garayzábal, 2019). A relative low proportion of Addition processes in Down syndrome had been previously reported by Van Borsel (1996), which could be explained by reduced short-term memory span. In the DS group, accumulated frequency of assimilation, omission and addition is still high (7.6/100 types) compared to the TD group (2.5/100 types), which might indicate that both groups are at different stages of late phonological development (Diez-Itza et al., 2001). As expected, relative frequency of processes in DS and TD groups showed similar qualitative profiles.

Effects of elicitation methods arose at class level, indicating higher sensitivity of the articulation test to substitution and to addition. This may be explained by target word types in the AT having more complex features, such as length or low frequency. Inversely, spontaneous speech favors the use of active vocabulary and less complex words, i.e., shorter and more familiar. As it would be expected in that case, individuals in the DS group were more adversely affected by increasing complexity of targets. Yousif (2018) found the inverse effects of elicitation methods which may be related to the method of counting, which was reduced to word types in the present study. The TD group showed an increased tendency to addition when assessed with the AT, which would not be expected in the DS group especially prone to reduce the length of words. In fact, results revealed an effect of elicitation method on omission, with spontaneous speech showing higher sensitivity to omission in the DS group. In this case, short unstressed function words, phonotactically placed in medial position in connected speech, pose additional difficulties to individuals in the DS group.

Position at syllable-level was also a source of variability. Percentage of full match (FM) was significantly higher in the DT group. However, differences were neutralized at the complex onset position. Pérez et al. (2018) observed lower full matches of tautosyllabic consonant clusters (complex onsets) in 5-year-olds with protracted phonological development (PPD) than children and adolescents with DS in the present study. On the other hand, they report for Chilean TD children from between 3 and 5 years of age match percentages averaging 60–70%, which is consistent with the percentages observed in the present study. In turn, Vergara et al. (2020) found higher matching percentages in Chilean TD 4-year-olds. Results on medial codas can be compared with percentage of matches in heterosyllabic groups (C1 being a medial coda) from the study by Bernhardt et al. (2015): TD children between 3 and 5 years of age showed FM percentages ranging 85–100%, while FM percentages in children with PPD of similar ages ranged 65–80% in line with findings in the present study. However, previous comparisons of phonological profiles of DS with those of children with phonological disorders yielded mixed results: Parsons and Iacono (1992) claimed that phonological processes did not differ from other phonologically-impaired populations, while inconsistent production of words appeared to be different from children with phonological disorder in the study by Dodd and Thompson (2001).

Concerning the debate on delayed vs. disordered phonological profiles in Down syndrome, results of the present study might be diversely interpreted. From the normalcy approach, the absence of outliers in the relative distribution of processes could be interpreted as indicative of simply delayed developmental trajectories (Schaner-Wolles, 2004). However, a more in-depth analysis might reveal undetected differences (e.g., complex onsets as in Vergara et al., 2020). From the neuroconstructivist approach, the focus should be placed on the developmental process itself (Thomas and Karmiloff-Smith, 2003). Thus, different rates of development at different linguistic levels may indicate distinct pathways or as suggested by Levy and Eilam (2013): asynchronous means atypical.

Atypically increased frequency of processes raises the question of unintelligibility (Kumin, 2006), which is also common to other neurodevelopmental disorders (Barnes et al., 2009). Results of the present study suggest low levels of speech accuracy in children and adolescents with DS, and therefore a need to assess and treat speech comprehensibility as a functional outcome of language intervention (Yoder et al., 2016a,b). Speech and motor-speech disorders are also present in some populations with acquired or degenerative impairments (e.g., Traumatic brain injury, Cerebrovascular accident or Dementia), affecting communication and social cognition (Geraci et al., 2010). Assessing phonological profiles might thus be important to plan specific speech interventions in the context of rehabilitative neuropsychology.

The limits of this study come primarily from no controlled individual differences that could account for significant percentages of the variance observed, especially in individuals with Down syndrome. A larger number of participants would have been necessary to minimize these differences, especially in the subgroups with Down syndrome. Nevertheless, this was a small-scale exploratory study and confidence in the conclusions drawn from the results is enhanced by effect size and statistical power estimates. Particularly, individual differences might have affected the results on the effect of elicitation methods given that different groups were assessed with different methods. Future research focused primarily on the effects of methods of elicitation should include intra-group designs. While articulation tests provide more controlled context for the assessment, it is always necessary to refine analyses in spontaneous speech, which is more valid and naturalistic but more difficult to conduct (Tomasello and Stahl, 2004). Phonological analysis of processes was limited to broad classes and percentages of full match in some positions at syllable level. A more in-depth study would be needed to assess specific characteristics and processes. To this end, the Phon program could provide detailed automated analyses.

## Data Availability Statement

The raw data supporting the conclusions of this article will be made available by the authors, without undue reservation.

## Ethics Statement

The studies involving human participants were reviewed and approved by Comité de Ética en la Investigación de la Universidad de Oviedo. Written informed consent to participate in this study was provided by the participants' legal guardian/next of kin.

## Author Contributions

ED-I had a primary role in the conception and design of the study, in the development of the coding scheme, in data analysis and discussion, and in drafting the manuscript. PV, MB, MM, and VM helped with the design and conducted research, carried out transcription, coding and data analyses, and helped draft the manuscript. All authors contributed to the article and approved the submitted version.

## Conflict of Interest

The authors declare that the research was conducted in the absence of any commercial or financial relationships that could be construed as a potential conflict of interest.

## References

[B1] Baddeley A. D. Jarrold C. (2007). Working memory and Down syndrome. J. Intellect. Disabil. Res. 51, 925–931. 10.1111/j.1365-2788.2007.00979.x 17990999

[B2] Barnes E. Roberts J. Long S. H. Martin G. E. Berni M. C. Mandulak K. C.. (2009). Phonological accuracy and intelligibility in connected speech of boys with fragile X syndrome or Down syndrome. J. Speech Lang. Hear. Res. 52, 1048–1061. 10.1044/1092-4388(2009/08-0001) 19641081 PMC2719827

[B3] Bellugi U. Lichtenberger L. Jones W. Lai Z. St. George M. (2000). The neurocognitive profile of Williams Syndrome: a complex pattern of strengths and weaknesses. J. Cogn. Neurosci. 12, 7–29. 10.1162/089892900561959 10953231

[B4] Bernhardt B. Mendoza E. Carballo G. Pérez D. Ávila C. Fresneda D.. (2016). Prueba de Fonologí a en Español [Phonology test for Spanish]. Vancouver, BC: School of Audiology and Speech Sciences, The University of British Columbia. Available online at: https://phonodevelopment.sites.olt.ubc.ca (accessed April 23, 2021).

[B5] Bernhardt B. M. Hanson R. Perez D. Avila C. Lleo C. Stemberger J. P.. (2015). Word structures of Granada Spanish-speaking preschoolers with typical versus protracted phonological development. Int. J. Lang. Commun. Disord. 50, 298–311. 10.1111/1460-6984.12133 25521065

[B6] Bernhardt B. M. Stemberger J. P. (2017). Investigating typical and protracted phonological development across languages, in Crosslinguistic Encounters in Language Acquisition: Typical and Atypical Development, eds E. Babatsouli, D. Ingram, and N. Mueller (Bristol: Multilingual Matters), 71–108.

[B7] Channell M. M. Thurman A. J. Kover S. T. Abbeduto L. (2014). Patterns of change in nonverbal cognition in adolescents with Down syndrome. Res. Dev. Disabil. 35, 2933–2941. 10.1016/j.ridd.2014.07.014 25112795 PMC4155014

[B8] Chapman R. (2006). Language learning in Down syndrome: the speech and language profile compared to adolescents with cognitive impairment of unknown origin. Down Syndr. Res. Pract. 10, 61–66. 10.3104/reports.306 16869363

[B9] Chapman R. Hesketh L. (2001). Language, cognition, and short-term memory in individuals with Down syndrome. Down Syndr. Res. Pract. 7, 1–7. 10.3104/reviews.108 11706807

[B10] Christodoulou C. (2015). Morphosyntactic illusions in Down syndrome: the role of phonetics and phonology, in BUCLD 39: Proceedings of the 38th Boston University Child Language Development—Online Supplement, eds E. Grillo, K. Jepson, and M. LaMendola (Boston: Boston University), 1–21. Available online at: http://www.bu.edu/bucld/supplementvol39/ (accessed April 23, 2021).

[B11] Diez-Itza E. Martínez V. Cantora R. Justicia F. Bosch L. (2001). Late phonological processes in the acquisition of Spanish, in Research on Child Language Acquisition, eds M. Almgren, A. Barreña, M. J. Ezeizabarrena, I. Idiazábal, and B. MacWhinney (Somerville, MA: Cascadilla Press), 790–799.

[B12] Diez-Itza E. Miranda M. Pérez V. Martínez V. (2019). Profiles of grammatical morphology in Spanish-speaking adolescents with Williams Syndrome and Down, in Atypical Language Development in Romance Languages, eds E. Aguilar-Mediavilla, L. Buil-Legaz, R. López-Penadés, V.A. Sánchez-Azanza, and D. Adrover-Roig (Amsterdam: John Benjamins), 219–234.

[B13] Dodd B. (1976). A comparison of the phonological systems of mental age matched, normal, severely subnormal, and Down's syndrome children. Int. J. Lang. Commun. Disord. 11, 27–42. 10.3109/13682827609011289 132957

[B14] Dodd B. Thompson L. (2001). Speech disorder in children with Down's syndrome. J. Intellect. Disabili. Res. 45, 308–316. 10.1046/j.1365-2788.2001.00327.x 11489052

[B15] Edwards J. Beckman M. E. (2008). Methodological questions in studying consonant acquisition. Clin. Linguist. Phonet. 22, 937–956. 10.1080/02699200802330223 19031192 PMC2728799

[B16] Geraci A. Surian L. Ferraro M. Cantagallo A. (2010). Theory of mind in patients with ventromedial or dorsolateral prefrontal lesions following traumatic brain injury. Brain Inj. 24, 978–987. 10.3109/02699052.2010.487477 20545452

[B17] Grieco J. Pulsifer M. Seligsohn K. Skotko B. Schwartz A. (2015). Down syndrome: cognitive and behavioral functioning across the lifespan. Am. J. Med. Genet. Part C Semin. Med. Genet. 169, 135–149. 10.1002/ajmg.c.31439 25989505

[B18] Hedlund G. Rose Y. (2019). PHON 3.0 [Computer Software]. The TalkBank System: Phon Project. Available online at: https://phon.ca (accessed April 23, 2021).

[B19] Hidalgo de la Guía I. Garayzábal E. (2019). Diferencias fonológicas entre síndromes del neurodesarrollo: evidencias a partir de los procesos de simplificación fonológica más frecuentes [Phonological differences between syndromes with neurodevelopmental disorders: evidence from the most frequent phonological processes]. J. Res. Speech Therapy 9, 81–106. 10.5209/rlog.62942

[B20] International Phonetic Association (2018). International Phonetic Alphabet and the IPA Chart. Available online at: http://www.internationalphoneticassociation.org/content/ipa-chart (accessed April 23, 2021).

[B21] Iverson J. M. Longobardi E. Caselli M. C. (2003). Relationship between gestures and words in children with Down's syndrome and typically developing children in the early stages of communicative development. Int. J. Lang. Commun. Disord. 38, 179–197. 10.1080/1368282031000062891 12745936

[B22] Jackson-Maldonado D. Galeote M. Flores-Guerrero M. F. (2019). The relationship between the lexicon and grammar in Spanish-speaking children with Down syndrome, in Atypical Language Development in Romance Languages, eds E. Aguilar-Mediavilla, L. Buil-Legaz, R. López-Penadés, V.A. Sánchez-Azanza, and D. Adrover-Roig (Amsterdam: John Benjamins), 219–234.

[B23] Jarrold C. Baddeley A. D. Hewes A. K. (2000). Verbal short-term memory deficits in Down syndrome: a consequence of problems in rehearsal? J. Child Psychol. Psychiatry 41, 233–244. 10.1017/S0021963099005120 10750549

[B24] Kent R. D. Vorperian H. K. (2013). Speech impairment in Down syndrome: a review. J. Speech Lang. Hear. Res. 56, 178–210. 10.1044/1092-4388(2012/12-0148) 23275397 PMC3584188

[B25] Kumin L. (2006). Speech intelligibility and childhood verbal apraxia in children with Down syndrome. Downs Syndr. Res. Pract. 10, 10–22. 10.3104/reports.301 16869369

[B26] Laws G. Gunn D. (2004). Phonological memory as a predictor of language development in children with Down syndrome: a five year follow up study. J. Child Psychol. Psychiatry 45, 326–337. 10.1111/j.1469-7610.2004.00224.x 14982246

[B27] Lenhard W. Lenhard A. (2016). Calculation of Effect Sizes. Dettelbach: Psychometrica. Available online at: https://www.psychometrica.de/effect_size.html (accessed April 23, 2021).

[B28] Levy Y. Eilam A. (2013). Pathways to language: a naturalistic study of children with Williams syndrome and children with Down syndrome. J. Child Lang. 40, 106–138. 10.1017/S0305000912000475 23217293

[B29] MacWhinney B. (2000). The CHILDES Project: Tools for Analyzing Talk. Mahwah, NJ: Lawrence Erlbaum Associates.

[B30] Martin G. E. Klusek J. Estigarribia B. Roberts J. E. (2009). Language characteristics of individuals with Down syndrome. Top. Lang. Disord. 29, 112–132. 10.1097/TLD.0b013e3181a71fe1 20428477 PMC2860304

[B31] Masterson J. J. Bernhardt B. H. Hofheinz M. K. (2005). A comparison of single words and conversational speech in phonological evaluation. Am. J. Speech Lang. Pathol. 14, 229–241. 10.1044/1058-0360(2005/023) 16229674

[B32] Morrison J. A. Shriberg L. D. (1992). Articulation testing versus conversational speech sampling. J. Speech Lang. Hear. Res. 35, 259–273. 10.1044/jshr.3502.259 1573866

[B33] Næss K. A. B. Lyster S. A. H. Hulme C. Melby-Lervåg M. (2011). Language and verbal short-term memory skills in children with Down syndrome: a meta-analytic review. Res. Dev. Disabil. 32, 2225–2234. 10.1016/j.ridd.2011.05.014 21628091

[B34] Parsons C. L. Iacono T. A. (1992). Phonological abilities of individuals with Down syndrome. Aust. J. Hum. Commun. Disord. 20, 31–45. 10.3109/asl2.1992.20.issue-2.03

[B35] Pérez D. Vivar P. Bernhardt B. M. Mendoza E. Ávila C. Carballo G.. (2018). Word-initial rhotic clusters in Spanish-speaking preschooler in Chile and Granada, Spain. Clin. Linguist. Phonet. 32, 481–505. 10.1080/02699206.2017.1359852 28956653

[B36] Roberts J. Long S. H. Malkin C. Barnes E. Skinner M. Hennon E. A.. (2005). A Comparison of phonological skills of boys with Fragile X syndrome and Down syndrome. J. Speech Lang. Hear. Res. 48, 980–995. 10.1044/1092-4388(2005/067) 16411789

[B37] Roberts J. E. Chapman R. Warren S. (eds.). (2008). Speech and Language Development and Intervention in Down Syndrome and Fragile X Syndrome. Baltimore: Brookes Publishing.

[B38] Roberts J. E. Price J. Malkin C. (2007). Language and communication development in Down syndrome. Ment. Retard. Dev. Disabil. Res. Rev. 13, 26–35. 10.1002/mrdd.20136 17326116

[B39] Rose Y. MacWhinney B. (2014). The PhonBank project: data and software-assisted methods for the study of phonology and phonological development, in The Oxford Handbook of Corpus Phonology, eds J. Durand, U. Gut, and G. Kristoffersen (Oxford: Oxford University Press), 308–401.

[B40] Rupela V. Manjula R. Velleman S. L. (2010). Phonological processes in Kannada-speaking adolescents with Down syndrome. Clin. Linguist. Phonet. 24, 431–450. 10.3109/02699200903450164 20136507

[B41] Schaner-Wolles C. (2004). Spared domain-specific cognitive capacities? syntax and morphology in Williams syndrome, in Williams Syndrome Across Languages, eds S. Bartke and J. Siegmüller (Amsterdam: John Benjamins), 93–124.

[B42] Shriberg L. D. Kwiatkowski J. (1980). Natural Process Analysis: A Procedure for Phonological Analysis of Continuous Speech Samples. New York, NY: Macmillan.

[B43] Shriberg L. D. Kwiatkowski J. (1985). Continuous speech sampling for phonologic analyses of speech-delayed children. J. Speech Hear. Disord. 50, 323–334. 10.1044/jshd.5004.323 4057974

[B44] Sommers R. K. Patterson J. P. Wildgen P. L. (1988). Phonology of Down syndrome speakers, ages 13–22. J. Child. Commun. Disord. 12, 65–91. 10.1177/152574018801200106

[B45] Stoel-Gammon C. (1980). Phonological analysis of four Down's syndrome children. Appl. Psycholinguist. 1, 31–48. 10.1017/S0142716400000710

[B46] Stoel-Gammon C. (2001). Down syndrome phonology: development patterns and intervention strategies. Down Syndr. Res. Pract. 7, 93–100. 10.3104/reviews.118 11721538

[B47] Thomas M. S. C. Karmiloff-Smith A. (2003). Modeling language acquisition in atypical phenotypes. Psychol. Rev. 110, 647–682. 10.1037/0033-295X.110.4.647 14599237

[B48] Tomasello M. Stahl D. (2004). Sampling children's spontaneous speech: how much is enough? J. Child Lang. 31, 101–121. 10.1017/S0305000903005944 15053086

[B49] Van Borsel J. (1988). An analysis of the speech of five Down's syndrome adolescents. J. Commun. Disord. 21, 409–421. 10.1016/0021-9924(88)90026-3 2972757

[B50] Van Borsel J. (1996). Articulation in Down's syndrome children. Eur. J. Disord. Commun. 31, 415–444. 10.3109/13682829609031330 9059573

[B51] Vergara P. Bernharndt B. M. Pérez D. Diez-Itza E. (2020). Consonant cluster acquisition in Chilean children with typical and protracted phonological development. Clin. Linguist. Phonet. 29, 1–19. 10.1080/02699206.2020.1851306 33251868

[B52] Vicari S. Caselli M. C. Tonucci F. (2000). Asynchrony of lexical and morphosyntactic development in children with Down syndrome. Neuropsychologia 38, 634–644. 10.1016/S0028-3932(99)00110-4 10689040

[B53] Vicari S. Marotta L. Carlesimo G. A. (2004). Verbal short-term memory in Down's syndrome: an articulatory loop deficit? J. Intellect. Disabil. Res. 48, 80–92. 10.1111/j.1365-2788.2004.00478.x 14723651

[B54] Yoder P. J. Camarata S. Woynaroski T. (2016a). Treating speech comprehensibility in students with Down syndrome. J. Speech Lang. Hear. Res. 59, 446–459. 10.1044/2015_JSLHR-S-15-0148 27300156 PMC4972011

[B55] Yoder P. J. Woynaroski T. Camarata S. (2016b). Measuring speech comprehensibility in students with Down syndrome. J. Speech Lang. Hear. Res. 59, 460–467. 10.1044/2015_JSLHR-S-15-0149 27299989 PMC4972012

[B56] Yousif N. S. (2018). Phonological development in children with Syndrome: an analysis of patterns and intervention strategies (Unpublished doctoral dissertation). University of Reading, Reading, England.

